# Construction of a Genome-Scale Metabolic Model of *Arthrospira platensis* NIES-39 and Metabolic Design for Cyanobacterial Bioproduction

**DOI:** 10.1371/journal.pone.0144430

**Published:** 2015-12-07

**Authors:** Katsunori Yoshikawa, Shimpei Aikawa, Yuta Kojima, Yoshihiro Toya, Chikara Furusawa, Akihiko Kondo, Hiroshi Shimizu

**Affiliations:** 1 Department of Bioinformatic Engineering, Graduate School of Information Science and Technology, Osaka University, 1–5 Yamadaoka, Suita, Osaka 565–0871, Japan; 2 Core Research for Evolutional Science and Technology, Japan Science and Technology Agency, 3–5 Sanbancho, Chiyoda-ku, Tokyo 102–0075, Japan; 3 Department of Chemical Science and Engineering, Graduate School of Engineering, Kobe University, 1–1 Rokkodai, Nada-ku, Kobe 657–8501, Japan; 4 Quantitative Biology Center, RIKEN, 6-2-3 Furuedai, Suita, Osaka 565–0874, Japan; The George Washington University, UNITED STATES

## Abstract

*Arthrospira* (*Spirulina*) *platensis* is a promising feedstock and host strain for bioproduction because of its high accumulation of glycogen and superior characteristics for industrial production. Metabolic simulation using a genome-scale metabolic model and flux balance analysis is a powerful method that can be used to design metabolic engineering strategies for the improvement of target molecule production. In this study, we constructed a genome-scale metabolic model of *A*. *platensis* NIES-39 including 746 metabolic reactions and 673 metabolites, and developed novel strategies to improve the production of valuable metabolites, such as glycogen and ethanol. The simulation results obtained using the metabolic model showed high consistency with experimental results for growth rates under several trophic conditions and growth capabilities on various organic substrates. The metabolic model was further applied to design a metabolic network to improve the autotrophic production of glycogen and ethanol. Decreased flux of reactions related to the TCA cycle and phosphoenolpyruvate reaction were found to improve glycogen production. Furthermore, *in silico* knockout simulation indicated that deletion of genes related to the respiratory chain, such as NAD(P)H dehydrogenase and cytochrome-c oxidase, could enhance ethanol production by using ammonium as a nitrogen source.

## Introduction

In recent years, bioproduction of fuels and chemicals from biomass has been intensively investigated to achieve a sustainable society. Cyanobacteria have also attracted increasing attention as biomass feedstock and host organisms for bioproduction because they can produce biomass and biofuels from CO_2_ as a carbon source and light as an energy source through photosynthesis [1]. Moreover, cyanobacteria have some advantages compared with higher plants, e.g., higher photosynthesis efficiency and lack of competition with food and land resources. To date, various products have been successfully produced using cyanobacteria, such as ethanol [2, 3], lactate [4], isobutanol [5], glycogen [6], and fatty acids [7].

*Arthrospira* (*Spirulina*) *platensis*, a filamentous non-N_2_-fixing cyanobacterium, is promising for use as a biomass feedstock because of its capability to accumulate a large amount of glycogen, which is an excellent feedstock for biofuel production [8, 9]. Under optimal light intensity and nitrate concentration, the intracellular glycogen content was reported to be up to 70% of the dry cell weight in *A*. *platensis* [6, 10]. Moreover, *A*. *platensis* has been produced as a superior nutrient because of its high content of protein and carotenes [11], and it is the most industrially cultivated microalgal species [12]. Its superior characteristics such as high pH tolerance and high salt tolerance [11] could prevent contamination by other organisms in outdoor cultivation.

Recently, metabolic simulation using a genome-scale metabolic model has been widely used for rational metabolic design for the improvement of target molecule production [13, 14]. A genome-scale metabolic model is an *in silico* metabolic model that includes most of the metabolic reactions and metabolites of the target strain [15, 16]. Flux balance analysis (FBA) enables the simulation of the metabolic flux distribution in the whole metabolic reactions of the metabolic model with the following two assumptions: steady-state metabolism and maximization of cell growth [17, 18]. This method can be applied to analyze the effects of gene manipulation and culture conditions on the metabolic flux distribution, and various studies have reported successful improvement of target production [13, 14]. In cyanobacteria, several genome-scale metabolic models have been constructed, such as those for *Synechocystis* sp. PCC 6803 [19–22], *Synechococcus* sp. PCC 7002 [23], and *Cyanothece* sp. ATCC 51142 [24], thus providing a detailed understanding of cyanobacterial metabolism. In the case of *A*. *platensis*, two small metabolic models were constructed that include 22 metabolic reactions [25] and 121 metabolic reactions [26]. Recently, a genome-scale metabolic model of *A*. *platensis* C1 (PCC9438) including 875 reactions was constructed, and metabolic phenotypes and essential genes under various culture conditions, such as autotrophic and mixotrophic conditions, were analyzed [27]. However, metabolic simulations to identify the candidate genes to be manipulated for improvement of target production have not been conducted.

In this study, we constructed a genome-scale metabolic model of *A*. *platensis* NIES-39 and developed metabolic engineering strategies to improve production of valuable materials, such as glycogen and ethanol. *A*. *platensis* NIES-39 is a well-studied *A*. *platensis* strain, as the whole genome sequence [28], glycogen production [6], and metabolome [29] have been analyzed. After construction of the metabolic model, simulation results using the constructed model were evaluated by comparison with the experimental results with regard to growth rates and growth capabilities on various organic substrates. Moreover, metabolic engineering strategies to improve autotrophic production of glycogen and ethanol were developed using the metabolic simulation.

## Materials and Methods

### 2.1. Construction of the genome-scale metabolic model of *A*. *platensis* NIES-39

A genome-scale metabolic model of *A*. *platensis* NIES-39 was constructed based on various information sources such as databases, the literature, and genome sequences. The annotation data were collected from CyanoBase [30], Kyoto Encyclopedia of Genes and Genomes (KEGG) [31], and the literature [28], and a draft metabolic model was constructed. To fill in the missing reactions of the draft model, candidate genes were identified from the genome sequence of *A*. *platensis* NIES-39 by comparison with the protein sequence of related cyanobacteria species such as *Synechocystis* sp. PCC 6803 and *Synechococcus* sp. PCC 7002 using BLASTP in KEGG (E-Value < 10^−10^) (http://www.genome.jp/tools/blast/). When the candidate genes for the synthesis reactions for the biomass components were not identified in the genome sequence, the reactions were added to the draft model based on the KEGG database. The cofactors of the reactions, such as NADPH and NADH, and transport reactions between the cytosol and periplasm and the periplasm and extracellular environment were added to the metabolic model based on previous genome-scale metabolic models of *Synechocystis* sp. PCC 6803 [29] and *Escherichia coli* [32].

The biomass components of *A*. *platensis* were collected from various previous reports. The macromolecular portion of the biomass was composed of 68% proteins, 16% carbohydrates, 0.88% DNA, 3.12% RNA, 11% lipids, and 1% chlorophyll, based on a previous study [26]. The amino acid composition in the proteins and the lipid composition were obtained from the literature [11, 33]. The carbohydrates were composed of glycogen [29], lipopolysaccharides [11], and polyhydroxybutyrate [34]. Carotenoids [35], which are categorized as hydrocarbons, were also included in the carbohydrates. The residual fraction of the carbohydrates consisted of peptidoglycans. The composition of nucleic acids in DNA and RNA was assumed to be proportional to the frequency in the whole genome sequence, e.g., 44.3% G + C content [28], following other study [20]. Because the ATP demand for biomass production in *A*. *platensis* NIES-39 was unknown, that of *Synechocystis* sp. PCC 6803 under autotrophic condition [36] was used in this model. The export reactions for intracellular metabolites, such as ethanol, lactate, acetate, pyruvate, and formate, were added to the metabolic model because the production of these metabolites was identified in the present study and also reported by previous studies [10, 37].

Finally, the metabolic model of *A*. *platensis* NIES-39 was constructed, which includes 673 unique metabolites and 746 metabolic reactions including 652 intracellular reactions, 60 transport reactions and 34 exchange reactions. The details of all reactions, metabolites, and biomass compositions are summarized in S1–S3 Tables.

### 2.2. Flux balance analysis

The metabolic flux distribution on the genome-scale metabolic model of *A*. *platensis* NIES-39 was calculated using FBA [18, 32]. Briefly, a pseudo-steady state of the metabolism was assumed, and the maximum and minimum ranges of the flux for each reaction were defined. These constraints provided a feasible space for the flux distribution in the metabolic model. To obtain the optimal flux distribution in the feasible space, an objective function was introduced and a linear programming technique was applied. This problem is represented by the following equation:

$$\begin{array}{l} \text{maximize}:c^{T}\cdot v\\ \text{subject to}:S\cdot v=0\\ v_{\min}\leq v\leq v_{\max} \end{array}$$

where *S* represents the stoichiometric matrix of metabolites in metabolic reactions, and *v* indicates a vector of the flux for each metabolic reaction. The values *v*_min_ and *v*_max_ represent the minimum and maximum constraints of the flux for each reaction, and *c* is a vector that represents the objective function to be maximized or minimized. In this study, biomass production was used as the objective function to be maximized, with the assumption that cellular metabolism is self-organized to maximize growth rate.

Because linear programming problems can present multiple solutions with an identical value for the objective function, the flux variability of each reaction was calculated [38]. In brief, after the maximum biomass production was determined by FBA, each flux was maximized and minimized under the condition of maximum biomass production.

All simulations were performed using Matlab (Mathworks Inc. Natick, MA) with a linear programming solver, GLPK (GNU Linear Programming Kit, http://glpkmex.sourceforge.net/).

For FBA simulation, the following external metabolites were assumed to be freely transported through the cell membrane: CO_2_, O_2_, H_2_O, sulfate, and phosphate. Nitrate and ammonium were used as the nitrogen source. Unless otherwise stated, the nitrate uptake rate was set to be free, and the ammonium uptake rate was set to zero. For the multiple knockout simulations to screen gene knockouts which are effective to increase target productions, we simply iterated FBA using metabolic networks with all possible combinatorial knockouts (e.g., three gene knockouts).

To simulate the metabolic flux distribution under autotrophic conditions, the photon uptake rate was set to a given value, and the glucose uptake rate was set to zero. For heterotrophic conditions, the uptake rate of one of the organic substrates, such as glucose, was set to a given value, and the photon uptake rate was set to zero. For simplification, we assumed that all photons are used for photosynthesis and neglected the effect of excess light energy to dissipate.

### 2.3. Culture experiment

The detailed culture conditions of *A*. *platensis* NIES-39 were described in a previous study [6]. The cells were grown in 500 mL Erlenmeyer flasks containing 250 mL of SOT medium under continuous illumination using white fluorescent bulbs (Life look HGX and NHG; NEC Corporation, Tokyo, Japan) with agitation at 100 rpm. The SOT medium consisted of 16.8 g/L NaHCO_3_, 0.5 g/L K_2_HPO_4_, 2.5 g/L NaNO_3_, 1.0 g/L K_2_SO_4_, 1.0 g/L NaCl, 0.2 g/L MgSO_4_ 7H_2_O, 0.04 g/L CaCl_2_ 2H_2_O, 0.01 g/L FeSO_4_ 7H_2_O, 0.08 g/L Na_2_ EDTA, and 1.0 mL of A5 solution (2.86 g/L H_3_BO_3_, 2.5 g/L MnSO_4_ 7H_2_O, 0.222 g/L ZnSO_4_ 7H_2_O, 0.079 g/L CuSO_4_ 5H_2_O, and 0.021 g/L Na_2_MoO_4_ 2H_2_O). Pre-cultivated cells were grown under 50 μmol photons/m^2^/s at 30°C. In the log-growth phase, the pre-cultivated cells were harvested by filtration and inoculated into fresh SOT medium without nitrate at 0.15 g dry cell weight (DW)/L. The main culture was performed under 250 μmol photons/m^2^/s at 30°C. Before inoculation, cells were washed three times with nitrate-free SOT medium to prevent a change in nitrate concentration.

### 2.4. Analytical methods

The cell concentration was measured according to the optical density of the culture medium at 750 nm (OD_750_) by using a UVmini-1240 spectrophotometer (Shimadzu, Kyoto, Japan).

The glycogen concentration was measured following the method of a previous study [6]. Glycogen was extracted from the cells using a modification of a method from a previous study [39]. The culture broth was centrifuged and the cell pellet was hydrolyzed at 90°C for 90 min with 100 μL of 30% KOH (g/L). The sample was cooled on ice and mixed with 600 μL of cooled ethanol. After 1 h, the sample was centrifuged and mixed with 600 μL of cooled ethanol. This step was repeated three times. Then, the sample was dried at 80°C for 10 min and re-suspended in 100 μL of water. The supernatant of the centrifuged sample was subjected to glycogen measurement. Glycogen content was determined by high-performance liquid chromatography (HPLC) (Shimadzu, Kyoto, Japan) using a size exclusion HPLC column (OHpak SB-806 M HQ; Shodex, Tokyo, Japan) and a reflective index detector (RID-10A; Shimadzu, Kyoto, Japan). The column temperature was kept at 60°C, and the mobile phase, 0.1 M NaNO_3_, was run at 1 mL/min.

The organic acid concentrations were measured using a HPLC system (Shimadzu, Kyoto, Japan) equipped with an Aminex HPX-87H column (Bio-Rad, Hercules, CA, U.S.A.). During HPLC analysis, the column temperature was kept at 65°C; the mobile phase, 2 mM H_2_SO_4_, was run at 0.5 mL/min; and the eluent absorbance was detected at 210 nm.

The ethanol concentration was measured using a gas chromatograph (GC) system with a hydrogen flame ionization detector (7890A; Agilent Technologies, USA) and a Stabiliwax column (0.32-mm internal diameter, 60-m length, and 1-μm thickness; Restek Co., USA).

## Results and Discussion

### 3.1. Construction of the genome-scale metabolic model of *A*. *platensis* NIES-39

A genome-scale metabolic model of *A*. *platensis* NIES-39 was constructed based on information from genome sequences, databases such as KEGG [31] and CyanoBase [30], and previously published material. The draft metabolic model was constructed based on the metabolic reactions collected from the KEGG database. Biomass compositions were collected from several previous reports [11, 26, 29, 33, 34]. The draft model was manually revised based on the literature and genome sequence information. For example, cyanobacteria have been reported to possess an incomplete TCA cycle because of a lack of α-ketoglutarate dehydrogenase, which converts α-ketoglutarate to succinyl-CoA [40, 41], and α-ketoglutarate dehydrogenase also has not been identified in *A*. *platensis* NIES-39. However, a recent study reported that a TCA bypass pathway complements the lack of the reaction [42] that converts α-ketoglutarate to succinate via the following two reactions: the conversion of α-ketoglutarate to succinic semialdehyde by α-ketoglutarate decarboxylase, and the conversion of succinic semialdehyde to succinate by succinic semialdehyde dehydrogenase. In *A*. *platensis* NIES-39, the gene encoding succinic semialdehyde dehydrogenase has been already annotated (NIES39_L06120), but the gene encoding α-ketoglutarate decarboxylase has not been identified. The candidate gene for α-ketoglutarate decarboxylase was searched for in the *A*. *platensis* NIES-39 genome sequence by BLASTP (http://www.genome.jp/tools/blast/) using the amino acid sequence of SynPCC7002_A2770, which was identified as α-ketoglutarate decarboxylase in *Synechococcus* sp. PCC 7002 [42], as a query sequence. As result, NIES39_L06130 was identified with high reliability (E-value = 0, identity = 79%). Thus, the reactions for the TCA bypass pathway were introduced into the metabolic model. In other cases, missing reactions that were not identified in the genome sequence of *A*. *platensis* NIES-39 but that are required for synthesis of biomass components, e.g., methionine, serine, and fatty acid biosynthesis reactions, were added based on the information of the KEGG database. Because *A*. *platensis* has been reported to produce some metabolites such as ethanol, formate, lactate, and acetate [10, 37], the export reactions of these metabolites were added to the metabolic model. Other reactions identified by BLASTP are summarized in S1 Table.

Finally, the metabolic model of *A*. *platensis* NIES-39, which included 746 reactions and 673 metabolites, was constructed (Table 1). The detailed information for the metabolic model is summarized in S1 and S2 Tables, and the functional classification of the metabolic reactions is described in S3 Table.

**Table 1:** Characteristics of the reconstructed metabolic model of *A*. *platensis* NIES-39. The number of unique metabolites was calculated by considering the metabolites present in more than one compartment as a single metabolite.

Features		Number
*Genomic information*		
	Genome size (bp)	6,788,435
	No. of open reading frames (ORFs)	6,631
	No. of annotated genes	2,542
*Reconstructed metabolic model*		
Metabolites		673
Reactions		746
	Annotated reactions	644
	Transport reactions	60
	Exchange reactions	34
	No. of ORFs including in the model	620
	ORF coverage	9%

### 3.2. Evaluation of the metabolic simulation results using the constructed model

To evaluate the simulation results using the constructed model, the simulated specific growth rates under various conditions were compared with the experimental results. To analyze the growth rate under autotrophic conditions, the maximum specific growth rate of *A*. *platensis* NIES-39 was calculated to be 0.074 (/h) from culture results under various light intensity conditions [6]. The maximum CO_2_ uptake rate was estimated to be 3.1 mmol/gDW/h from the carbon composition in the biomass [43] and the maximum specific growth rate, with the assumption that the cells do not excrete any metabolites into the medium in the log growth phase. The specific growth rate under autotrophic conditions was simulated using the metabolic model with the following parameters: CO_2_ uptake rate, 3.1 mmol/gDW/h; photon uptake rate, free; glucose uptake rate, zero. The simulated specific growth rate (0.069/h) agreed well with the experimental results (0.074/h). The specific growth rate under heterotrophic conditions was also analyzed. The specific growth rate and specific glucose uptake rate were calculated from a previous experiment under heterotrophic conditions generated with glucose and without light illumination [37]. Metabolic simulation was performed to imitate the experimental conditions with the following parameters: glucose uptake, 0.16 mmol/gDW/h calculated from the experimental result [37]; photon uptake rate, zero. The simulated specific growth rate (0.11/h) was almost identical to the experimental result (0.10/h).

For further evaluation of the simulation results, growth capabilities on various organic substrates under heterotrophic conditions were analyzed. In a previous study, *A*. *platensis* NIES-39 was grown on 14 organic substrates, such as sugars, metabolites of the central metabolism, and amino acids, under heterotrophic conditions [44]. Note that *A*. *platensis* NIES-39 was described with its synonymous name, *Spirulina platensis* IAM M-135, in the previous study [44]. To simulate the growth capabilities of the metabolic model on these organic substrates, the uptake rate of each substrate was set to 10 mmol/gDW/h, and the photon uptake rate was set to zero. Because the uptake rate of each substrate was not measured in the experiment, the same uptake rate was set for each substrate for metabolic simulation, and only the growth capability was compared. The simulation results indicated that *A*. *platensis* could grow on all substrates and were consistent with the experimental results in 11 of 14 cases (Table 2). Inconsistencies between the simulation and experimental results were found in the case of α-ketoglutarate, succinate, and fumarate. To enable growth using α-ketoglutarate as a sole carbon source, a complete TCA cycle or glyoxylate shunt is required to supply the carbon for gluconeogenesis. However, the enzymes isocitrate lyase and malate synthase in the glyoxylate shunt and α-ketoglutarate dehydrogenase in the TCA cycle have not been identified in the genome sequence of *A*. *platensis* NIES-39. Recently, the TCA bypass pathway was determined to complement the incomplete TCA cycle in cyanobacteria [42]. *A*. *platensis* NIES-39 possesses enzymes highly homologous to those of the TCA bypass pathway and can grow on citrate and glutamate [44], which also requires a complete TCA cycle, thus suggesting the existence of a TCA bypass pathway. Therefore, it is possible that *A*. *platensis* NIES-39 lacks α-ketoglutarate uptake under the examined conditions because low uptake capability for α-ketoglutarate was reported in cyanobacteria [45]. Another possible reason for this result is that *A*. *platensis* NIES-39 does not possess a transporter for these metabolites because the transporter of α-ketoglutarate was annotated as a “putative” transporter (NIES39_G00660 gene) in *A*. *platensis* NIES-39. In fact, metabolic simulation without the α-ketoglutarate transport reaction predicted non-growth on α-ketoglutarate.

**Table 2:** Comparison of the growth capabilities on the various organic substrates.

Substrate	Experimental [44]	Simulation
Glucose	+	+
Maltose	+	+
Sucrose	+	+
Maltotriose	+	+
Pyruvate	+	+
Lactate	+	+
Citrate	+	+
α-Ketoglutarate	−	+
Succinate	−	+
Fumarate	−	+
Malate	+	+
Oxaloacetate	+	+
Glutamate	+	+
Aspartate	+	+

+ and − indicate growth or non-growth on the corresponding substrate, respectively, under heterotrophic conditions.

In the case of succinate and fumarate, the growth ability on citrate and glutamate suggests the existence of the activity of succinate dehydrogenase and fumarase required for utilization of succinate and fumarate in *A*. *platensis* NIES-39 (Table 2). The transporter of these metabolites was also annotated as being expressed from the same gene as the α-ketoglutarate transporter. Thus, this inconsistency may have resulted from a lack of existence of the transporter for these metabolites or a lack of their uptake under the examined conditions.

Overall, although further experimental validation and improvement of the metabolic model are required to eliminate these inconsistencies, the simulation results for the growth rate and growth capability are consistent with the experimental results, which suggests that the constructed metabolic model is reliable and could be applied for metabolic simulations of *A*. *platensis* NIES-39.

### 3.3. Glycogen production under nitrogen limitation conditions

**Fig 1:**
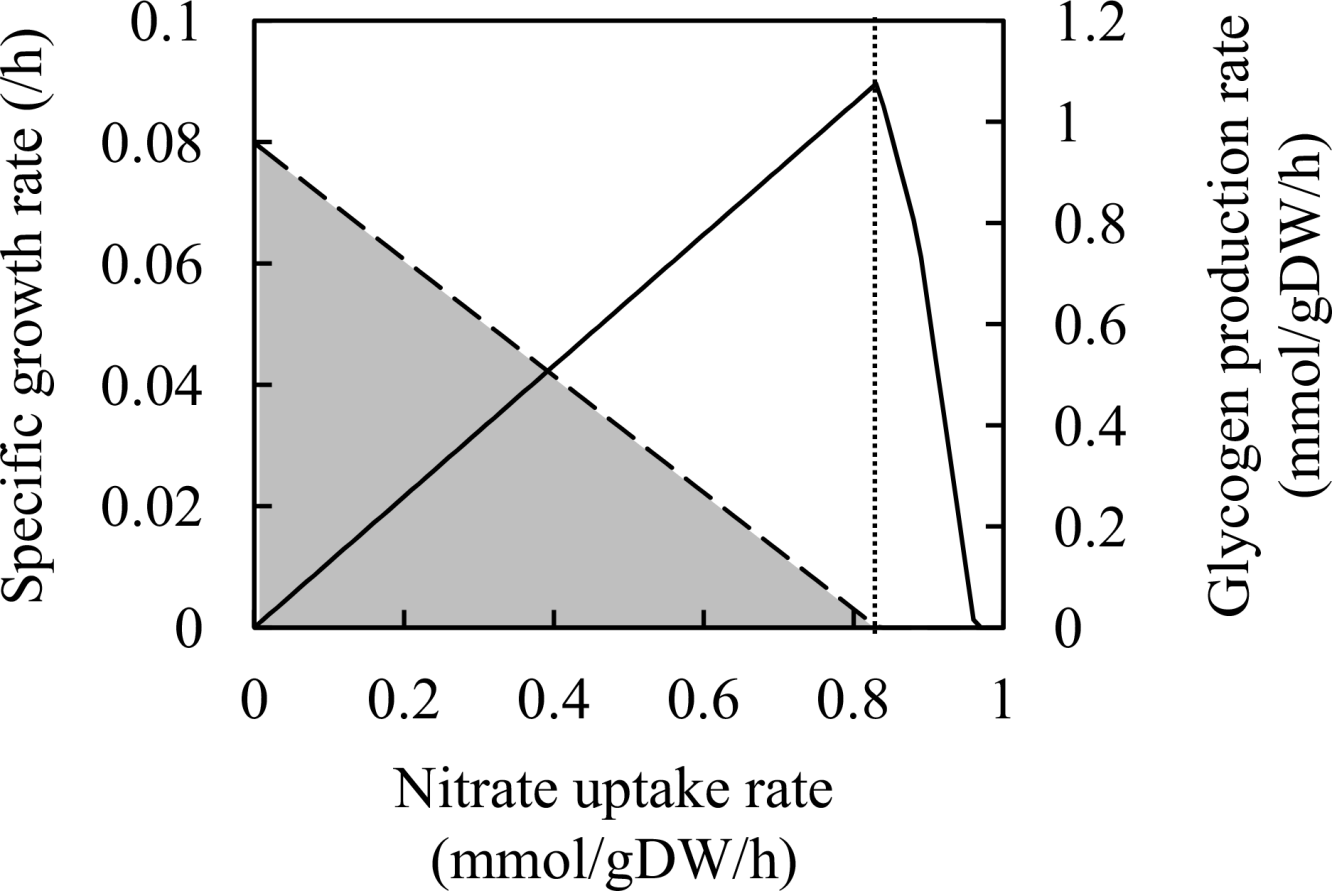
Effect of nitrate uptake rate on growth and glycogen production. The specific growth rate (solid line) and glycogen production rate (dashed line) at each nitrate uptake rate are shown. The dotted line indicates the optimal uptake rate for biomass production. The grey area represents the solution space of the glycogen production rate calculated by flux variability analysis [38], as the glycogen production rate was undetermined.

**Fig 2:**
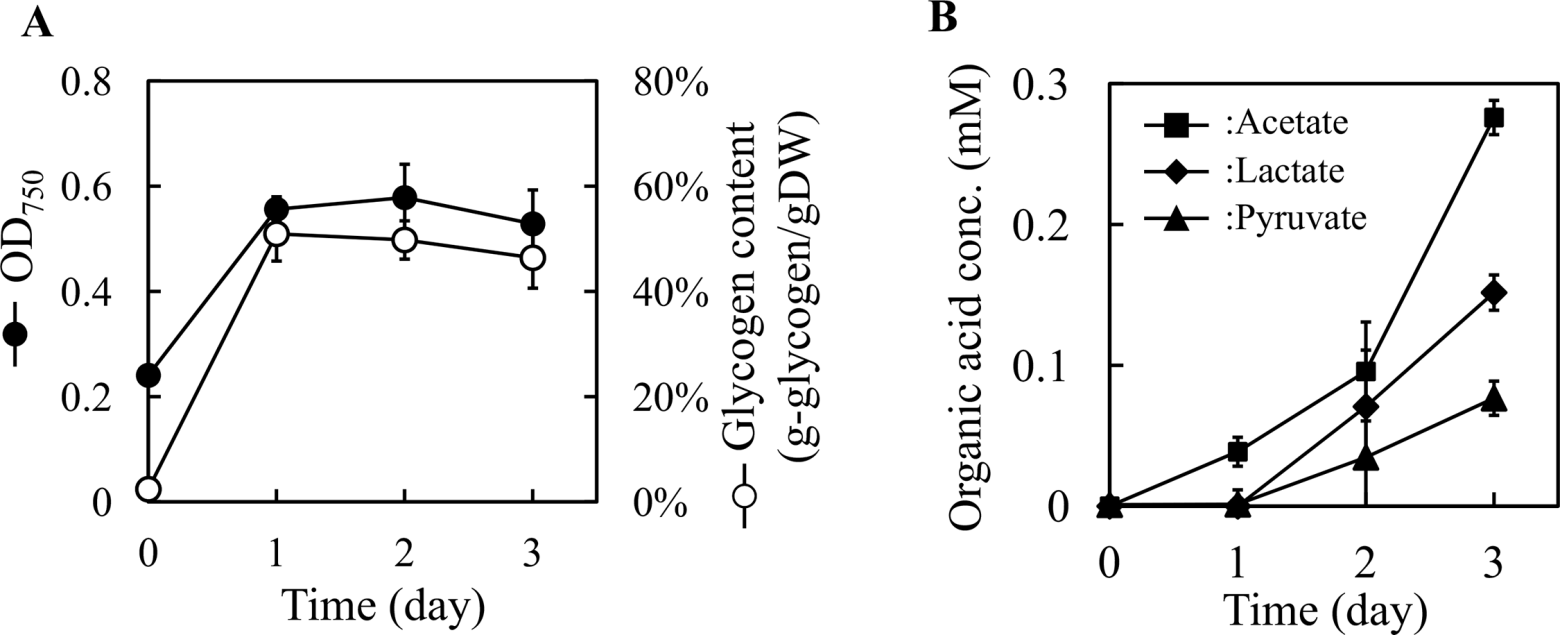
Culture profile under nitrogen depletion conditions. Growth and intracellular glycogen (A) together with the concentrations of organic acids in the culture medium (B) were summarized. Closed circle, OD_750_; open circle, glycogen content; square, acetate; diamond, lactate; triangle, pyruvate. Error bars represent the standard deviation of triplicate experiments.

Glycogen accumulation by nitrogen limitation was simulated using the metabolic model. *A*. *platensis* is a promising host strain for use as a biomass feedstock because of its ability to accumulate a large amount of glycogen [6]. Previous studies reported a drastic increase of intracellular glycogen contents by nitrogen depletion of the culture medium [6, 10, 29]. The effects of nitrogen limitation on metabolism under autotrophic conditions were analyzed using the metabolic model. A virtual reaction for transport of glycogen from the cytosol to the extracellular compartment was added to the metabolic model to simulate glycogen production. For simplification, glycogen is treated as a monomer (C6H10O5, MW = 162.141) in this model, where one mole of glycogen is generated from one mole of glucose-1-phosphate. Metabolic simulations were performed with the following parameters: photon uptake rate, 50 mmol/gDW/h; glucose uptake rate, zero. The effect of nitrogen limitation was analyzed by changing the nitrogen uptake rate. The biomass and glycogen production rates as functions of the nitrate uptake rate are shown in Fig 1. When the nitrate uptake rate was lower than the optimal uptake rate for biomass production, the biomass production rate decreased and the glycogen production rate increased as observed for the experimental results [6]. The mechanism underlying the increase of glycogen production is as follows. Limitation of nitrate uptake rate decreased the growth rate, and the excess carbon caused by growth limitation was balanced with glycogen production. However, the increase of glycogen production was only one of the feasible solutions, and production of other metabolites such as ethanol, lactate, acetate, pyruvate, and formate was also one of the solutions under nitrate limitation. To confirm the production of these metabolites, the extracellular metabolites in the culture medium under nitrate depletion were experimentally analyzed. *A*. *platensis* NIES-39 was cultured with nitrate-supplemented medium, and cells were transferred to the fresh medium without nitrate. The glycogen content was increased by nitrate depletion, and production of acetate, lactate, and pyruvate was identified as estimated from the simulation results (Fig 2). Fumarate, succinate, and ethanol were not identified from HPLC and GC. Based on these results, deletion of genes related to the biosynthesis of acetate and lactate could be a strategy to improve glycogen production using *A*. *platensis* under nitrate depletion conditions.

### 3.4. Design of a metabolic network for glycogen production

The metabolic model enabled us to develop metabolic engineering strategies for improvement of glycogen production. We performed an *in silico* multiple knockout simulation to identify the candidate genes to be deleted for improvement of glycogen production under autotrophic conditions with the following parameters: photon uptake rate, 50 mmol/gDW/h; glucose uptake rate, zero. All possible single, double, and triple gene knockout simulations were performed. Note that if several genes were assigned to one metabolic reaction in the model, knockout of one of these genes was used to knock out the reaction. Knockout simulations of more than four genes could not be performed because of the computational time required. However, a multiple knockout could not identify candidate genes to be deleted, possibly because glycogen production does not contribute to the improvement of biomass production, as glycogen biosynthesis is not accompanied by NAD(P)H production or consumption and requires ATP consumption, which reduces the growth rate.

**Fig 3:**
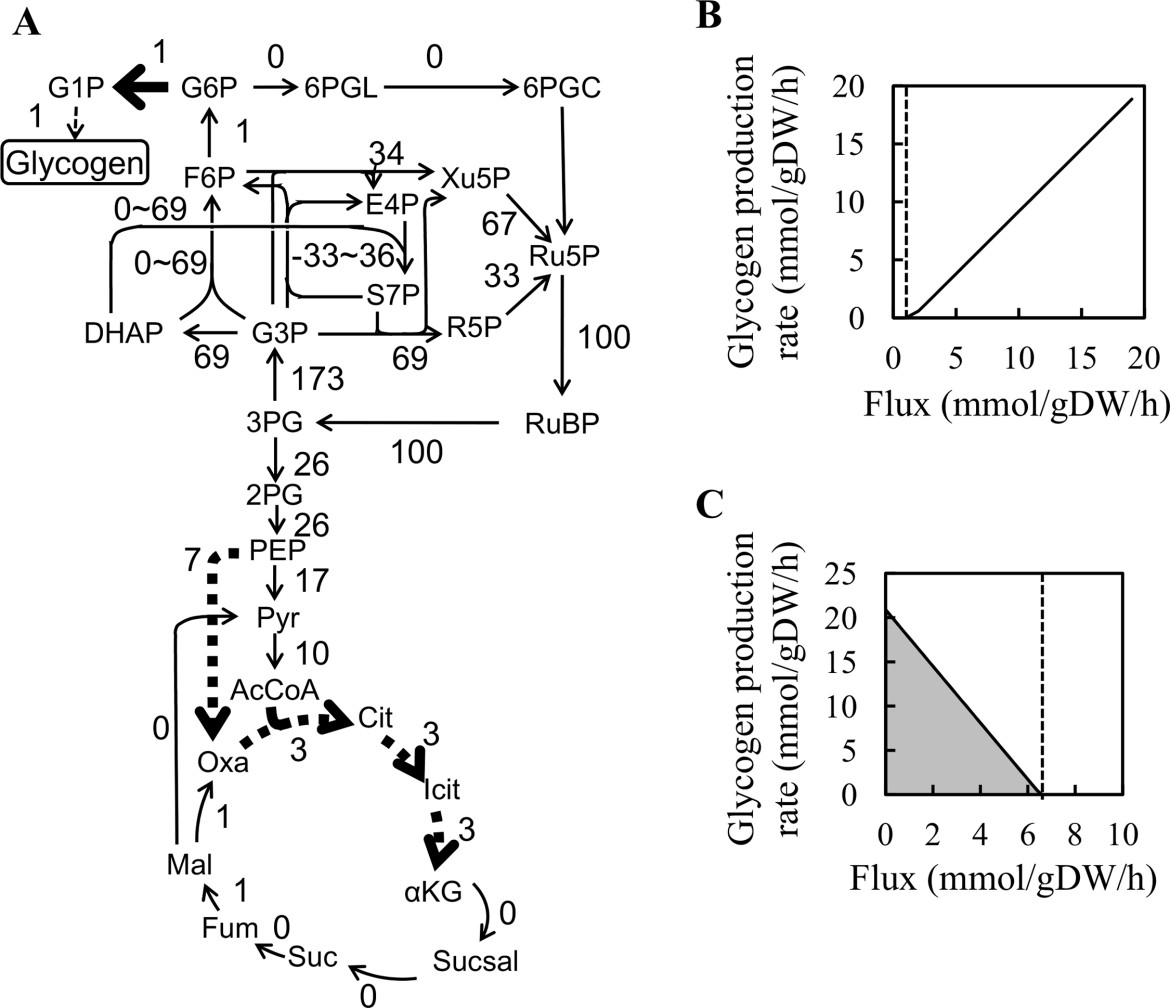
Flux response analysis for glycogen production. The simulated metabolic flux distribution under autotrophic conditions is shown (A). The values indicate the metabolic flux of each reaction. The flux was normalized to that of the reaction catalyzed by ribulose 1,5-bisphosphate carboxylase/oxygenase. Bold and dashed-bold arrows indicate the reactions whose activation or repression increased glycogen production, respectively, identified by flux response analysis. The reactions “DHAP + E4P ↔ S7P”, “DHAP + G3P ↔ F6P” and “G3P + S7P ↔ E4P + F6P” were undetermined, and the flux range of these reactions was calculated by flux variability analysis [38]. FRA results of the phosphoglucomutase reaction (G6P → G1P) (B) and phosphoenolpyruvate carboxylase reaction (PEP → Oxa) (C) are shown. The X-axis indicates the flux of the corresponding reaction and the Y-axis indicates the glycogen production rate excluding the flux for biomass production, as glycogen is a component of the biomass. The dotted line indicates the optimal flux of the corresponding reaction. The grey area in Fig 3(C) represents the solution space calculated by flux variability analysis. 2PG, glycerate-2-phosphate; 3PG, 3-phosphoglycerate; 6PGC, 6-phospho-gluconate; 6PGL, 6-phospho-glucono-1,5-lactone; AcCoA, acetyl-CoA; αKG, α-ketoglutarate; Cit, citrate; DHAP, dihydroxyacetone phosphate; E4P, erythrose-4-phosphate; F6P, fructose-6-phosphate; Fum, fumarate; G1P, glucose-1-phosphate; G3P, glyceraldehyde-3-phosphate; G6P, glucose-6-phosphate; Icit, isocitrate; Mal, malate; Oxa, oxalate; PEP, phosphoenolpyruvate; Pyr, pyruvate; R5P, ribose-5-phosphate; Ru5P, ribulose-5-phosphate; RuBP, ribulose-1,5-bisphosphate; S7P, sedoheptulose-7-phosphate; Suc, succinate; Sucsal, succinyl semialdehyde; Xu5P, xylulose-5-phosphate.

Next, we analyzed the effect of increasing or decreasing the flux of each reaction on glycogen production by flux response analysis (FRA) [14, 46]. In FRA, the changes of the target flux (glycogen production) are analyzed in response to artificial repression or activation of the flux of each reaction. FRA was performed with the following parameters: photon uptake rate, 50 mmol/gDW/h; glucose uptake rate, zero. Then, FBA was performed by changing the flux of each reaction related to the central metabolism, such as glycolysis, TCA cycle, pentose phosphate pathway (PPP), and the Calvin cycle. As expected, activation of the phosphoglucomutase reaction (G6P → G1P, abbreviations are described in figure legend of Fig 3), which produces a precursor of glycogen, enhanced glycogen production (Fig 3). Repression of reactions such as those mediated by phosphoenolpyruvate carboxylase (PEP → Oxa), citrate synthase (Oxa + AcCoA → Cit), aconitate hydratase (Cit → Icit), and isocitrate dehydrogenase (Icit → aKG), enhanced glycogen production, as decreased flux of these reactions repressed biomass production, and excess carbon was used for glycogen production.

An increase of the precursor supply is an important strategy to improve target molecule production. However, FRA estimated that glycogen production was not enhanced by increased flux of the phosphoglucose isomerase reaction (F6P → G6P). When the flux of phosphoglucose isomerase was increased, the flux into oxidative PPP was increased because increase of oxidative PPP flux contributes to biomass production rather than increase of glycogen production. When *zwf* encoding glucose-6-phosphate dehydrogenase was deleted, glycogen production was predicted to be increased by activation of the phosphoglucose isomerase reaction (data not shown). Under the nitrate depletion condition in *Synechocystis* sp. PCC 6803, in which the flux toward glycogen was increased via phosphoglucose isomerase, the intracellular concentration of 6-phosphogluconate belonging to oxidative PPP was increased, suggesting that the flux through PPP was increased [47]. Therefore, deletion of genes related to the oxidative PPP, such as *zwf*, is a possible strategy to improve glycogen production under nitrogen limitation conditions as predicted by metabolic simulation.

### 3.5. *In silico* knockout simulation for ethanol production

The metabolic model was further applied to design the metabolic network to improve the production of another useful product, ethanol, which has the potential to replace fossil fuels. *A*. *platensis* NIES-39 is a natural producer of ethanol [10]. Both NADH and NADPH dependent alcohol dehydrogenase reactions were included in the metabolic model, since the specificity of alcohol dehydrogenase was unknown. First, the triple gene knockout simulation was performed to identify the candidate genes to be deleted for improvement of ethanol production with following parameters: photon uptake rate, 50 mmol/gDW/h; glucose uptake rate, zero. Nitrate was used as the nitrogen source. In this analysis, all flux estimations were performed under the autotrophic condition, the purpose of this screening was to improve the production of ethanol from CO_2_ as a carbon source and light as an energy source, for bio-engineering purposes. As results of this screening, two sets of knockouts were identified (Tables 3 and 4). The details, such as the identified genes and reactions, are summarized in S4 Table. The mechanisms underlying the increased ethanol production in these strains are not clear, but the excess NAD(P)H might be generated by these knockouts and was balanced with ethanol production, i.e., through the alcohol dehydrogenase reaction. Although the candidates were identified, their estimated carbon-molar yield (C-mol%), which was calculated as the carbon ratio between ethanol production to fixed CO_2_, were very low (approximately 2 C-mol%). When nitrate was used as a nitrogen source, a large amount of excess NAD(P)H could not be produced because a great amount of NADPH was used to reduce the oxidized ferredoxin generated by ferredoxin-nitrate reductase, which reduced nitrate to ammonium. Therefore, we speculated that using ammonium rather than nitrate could reduce the oxidization of NADPH by nitrate reduction and generate excess NADPH.

**Table 3:** *In silico* knockout simulation for ethanol production with nitrate as nitrogen source. Detailed information is summarized in S4 Table.

Relative growth rate to wild type model.	Ethanol yield (C-mol%)	Knockout targets
96%	2%	NADH dehydrogenase
		NADPH dehydrogenase
		L-Valine:pyruvate aminotransferase
		Malic enzyme
97%	1%	NADH dehydrogenase
		NADPH dehydrogenase
		L-Valine:pyruvate aminotransferase
		Malate dehydrogenase

**Table 4:** *In silico* knockout simulation for ethanol production with ammonium as nitrogen source. Detailed information is summarized in S4 Table.

Relative growth rate to wild type model.	Ethanol yield (C-mol%)	Knockout targets
18%	55%	NADH dehydrogenase
		NADPH dehydrogenase
		Cytochrome-c oxidase
		Phosphoglycerate mutase
18%	55%	NADH dehydrogenase
		NADPH dehydrogenase
		Cytochrome-c oxidase
	
		Enolase
20%	54%	NADH dehydrogenase
		NADPH dehydrogenase
		Cytochrome-c oxidase
		Phosphoenolpyruvate synthase
37%	43%	NADH dehydrogenase
		NADPH dehydrogenase
		Cytochrome-c oxidase

The triple gene knockout simulation was performed using ammonium as a nitrogen source by changing the nitrate uptake rate to zero and the ammonium uptake rate to free. The knockout simulations identified several sets of knockout candidates whose deletion could improve ethanol production (Table 4 and S4 Table). The reactions of NADH dehydrogenase, NADPH dehydrogenase, and cytochrome-*c* oxidase, which belong to the respiratory chain, were commonly included among the candidates. NADH dehydrogenase and NADPH dehydrogenase are encoded by genes from NIES39_E02860, NIES39_A01530, NIES39_A08120, NIES39_K04850, NIES39_O01600, NIES39_O01620, NIES39_A06520, NIES39_K04840, NIES39_A06510, NIES39_O01610, NIES39_E02840, NIES39_O07110, NIES39_E02850, NIES39_A08140, NIES39_A08130, NIES39_F00440, NIES39_J01870, NIES39_H00330, NIES39_L04070, NIES39_L04060, and NIES39_L04040. Cytochrome-*c* oxidase is encoded by NIES39_O03120, NIES39_O03080, NIES39_O03090, NIES39_O03100, NIES39_A01920, and NIES39_A01910. The ethanol yield was estimated to be zero in the original metabolic model and 43 C-mol% in this knockout model. Because NAD(P)H was oxidized through the respiratory chain, excess NADPH was generated in the deletion model of these reactions. Generally, in non-photosynthetic microorganisms, ethanol production is performed under anaerobic conditions to generate excess intracellular NADH by reducing respiratory chain activity. However, in cyanobacteria, oxygen is produced by photosynthesis under autotrophic conditions, which makes it difficult to generate anaerobic conditions. Thus, disruption of the respiratory chain may be effective to increase ethanol production rather than anaerobic cultivation in cyanobacteria. In this case, the specificity of alcohol dehydrogenase for NADH and NADPH did not affect the results because transhydrogenase converts NADPH to NADH in our metabolic model. The disruption of respiratory chain also resulted in increasing flux on Photosystem I (PSI) and decreasing flux on Photosystem II (PSII). This change in PSI and PSII fluxes was probably due to compensating the decrease in proton pumping flux by the disruption of respiratory chain. Although the biomass production significantly decreased by the disruption of respiratory chain, still the cells can produce biomass. In the disrupted network, the proton pumping activity is maintained by the process of photosynthesis, which in turn results in producing ATP.

Moreover, additional deletions of some genes in the knockout of the respiratory chain were predicted to further increase ethanol production (Table 4), i.e. genes related to phosphoglycerate mutase, enolase and pyruvate kinase. Additional knockout of phosphoglycerate mutase encoded by NIES39_D07330, which converts 3-phosphoglycerate to 2-phosphoglycerate, resulted in a 55 C-mol% of ethanol yield. This knockout prevented the flux from 3-phosphoglycerate downstream of glycolysis, as 3-phosphoglycerate was converted to pyruvate via the serine biosynthesis pathway rather than glycolysis. In this strain, ethanol production was increased by balancing the excess NAD(P)H generated by following mechanisms: (1) NADH was generated by the serine biosynthesis pathway, and (2) the decreased growth rate reduced the usage of NAD(P)H for biomass formation because knockout of 3-phosphoglycerate prevented ATP production by pyruvate kinase. As for the deletion of enolase encoded by NIES39_B01040 and NIES39_C00710, the mechanism was the same as that in the knockout of phosphoglycerate mutase. In the case of the deletion of pyruvate kinase encoded by NIES39_M02310 and NIES39_P00240, the reduced ATP production resulting from knockout of pyruvate kinase decreased the growth rate, resulting in excess NAD(P)H.

## Concluding Remarks

In this study, we constructed a genome-scale metabolic model of *A*. *platensis* NIES-39 and developed novel metabolic engineering strategies for glycogen and ethanol production. The metabolic model was constructed based on information from databases and the literature, and the simulation results using the metabolic model were evaluated by comparing them with the experimental results for growth rates and growth capabilities on various organic carbon sources. The high consistency between the simulation and experimental results indicates the availability of the constructed metabolic model for metabolic simulation. The metabolic simulation was further applied to demonstrate glycogen production under nitrogen limitation conditions and to design metabolic engineering strategies to increase glycogen production by FRA. Moreover, *in silico* knockout simulation successfully identified candidate genes to be deleted for improvement of ethanol production.

*A*. *platensis* is expected to be a suitable feedstock and host strain for bioproduction because of its superior characteristics for industrial bioproduction processes. The genome-scale metabolic model constructed in this study will provide a detailed metabolic understanding and valuable information to achieve industrial bioproduction using *A*. *platensis*.

## Supporting Information

S1 Table Genome-scale metabolice model of A. platensis NIES-39. (XLSX)

S2 Table Metabolite list. (XLSX)

S3 Table Functional classification of metabolic reactions in the genome-scale metabolic model of *A*. *platensis*. (XLSX)

S4 Table *In silico* knockout simulation for ethanol production. (XLSX)
